# The Effect of Supramolecular Humic Acids on the Diffusivity of Metal Ions in Agarose Hydrogel

**DOI:** 10.3390/molecules27031019

**Published:** 2022-02-02

**Authors:** Martina Klučáková

**Affiliations:** Faculty of Chemistry, Brno University of Technology, Purkyňova 118/464, 612 00 Brno, Czech Republic; klucakova@fch.vutbr.cz; Tel.: +42-054-114-9410

**Keywords:** humic acids, diffusion, interaction, supramolecular, self-assembly, copper

## Abstract

Humic acids are known as natural substances of a supramolecular nature. Their self-assembly ability can affect the migration of heavy metals and other pollutants in nature. The formation of metal-humic complexes can decrease their mobility and bioavailability. This study focuses on metal ions diffusion and immobilization in humic hydrogels. Humic acids were purchased from International Humic Substances Society (isolated from different matrices—peat, soil, leonardite, water) and extracted from lignite mined in Czech Republic. Copper(II) ions were chosen as a model example of reactive metals for the diffusion experiments. The model of instantaneous planar source was used for experimental data obtained from monitoring the time development of copper(II) ions distribution in hydrogel. The effective diffusion coefficients of copper(II) ions showed the significant dependence on reaction ability of humic hydrogels. Lower amounts of the acidic functional groups caused an increase in the effective diffusion coefficient. In general, diffusion experiments seem to act as a valuable method for reactivity mapping studies on humic substances.

## 1. Introduction

Reactivity and transport properties of metal ions are important both for evaluating and understanding the role of humic acids in natural systems and human-driven applications in solving their structural questions. Humic acids are recognized as a component of natural organic matter that plays a key role in the self-detoxification of soils and sediments. Their self-assembly and complexation ability can result in the reduction of the mobility of toxic metal ions (and other pollutants), biological uptake and bioaccumulation of toxic chemicals in plants as well as the pollution of the underground water supplies [1,2,3,4,5].

The structure of humic acids is very complex. There are several hypothesis and models to prove this. Nowadays, the concept of humic acids having a supramolecular structure is widely accepted. This concept presumes that humic substances are associations of small molecules self-assembled by weak forces and hydrogen bonds [6,7,8,9,10,11,12,13]. Humic associations are formed by the self-organization of hydrophobic and amphiphilic compounds whereby hydrophilic structures are formed mainly by carbohydrate chains and aromatic rings, amphiphilic by ionizable functional groups [14,15]. Some results [13] showed that the associations have polar surfaces and unpolar cores, therefore polar surfaces of humic particles (containing by dissociable functional groups) are in contact with soil solution, less polar subunits are located in the inner layers, and unpolar structures are accommodated in the core of humic association, outward, towards the surrounding free soil environment. Other authors [11,16,17,18] stated that supramolecular associations and humic macromolecules can co-exist in the structure of humic acids. Many authors [1,10,12,18,19,20,21,22] confirmed that the conformational arrangement of humic acids can control their interactions with pollutants in nature without regard to their preferred model of humic structure. Our previous works [23,24,25,26] showed that the molecular organization of humic acids is strongly affected by their concentration. Changes in secondary structure were observed at concentrations around 0.02 g·dm^−3^ and 1 g·dm^−3^. Changes observed at lower concentration were attributed to the formation of particles of between 0.1 and 11 μm in size and changes in their hydration shells. Changes in the secondary structure of humic acids resulted in an increase in colloidal stability, a decrease in polydispersity, and the formation of humic aggregates with rigid structures were observed in systems with higher concentrations (>1 g·dm ^−3^) were detected. A similar break in humic properties at a certain humic content in the studied system was also observed in other works [8,27,28,29,30] and the high concentration region is often considered to be a pseudo-micellar organization phase [28].

The majority of published work is focused on humic acids in solutions. Studies of humic hydrogels are relatively scarce. In fact, mainly hydrogels based on humic substances in combination with other materials were investigated [31,32,33,34,35,36]. Humic substances were added into hydrogels based on starch [29,36], chitosan and poly (vinyl alcohol) [31], polyacrylamide [32] and agarose [30,34,35,37,38]. This work focuses on the hydrogels based on agarose enriched by humic acids. The simple method, based on the special accessory providing controlled fine vertical movement of the cuvette in the spectrophotometer providing concentration profiles of diffusing particles in hydrogel [35,38], was used. It was found that this method can be used in several experimental arrangements (diffusion couple, diffusion from instantaneous planar, source, diffusion from constant source, etc.). Simultaneously, the method can be used for the reactivity mapping of humic acids because the transport of metal ions (and other pollutants) in humic gels is strongly influenced by their reactivity [2,24,30,31,32,33,34,35,36,37,38,39,40]. Heavy metal ions can be bound to humic acids with different bond strengths. The results can thus provide the information on their mobility and (bio)availability. In this work, the method of the diffusion instantaneous planar source method [39,40,41,42,43,44] was used. Copper/II) ion as a traditional model ion was used because of its strong affinity to humic acid [1,2,39,40,42].

## 2. Results and Discussion

The basic characteristics of the humic samples used are listed in Table 1 [45]. It can be seen that the higher content of carbon has LEHA, and this sample also has the lowest content of hydrogen, oxygen and O/C ratio. In contrast, its C/H ratio is the highest. A very low O/C ratio, O content and high C content and C/H ratio were also determined for ESHA. The highest H and O contents were determined for LGHA and SRHA, accompanied by high O/C ratios. In contrast, the highest O content and O/C ratio were determined for NLHA. The content of acidic functional groups was highest for SRHA, the lowest for LGHA. Although the O/C ratio is considered as an indicator of the content of acidic functional groups, the total acidity does not fully correspond with it. The reason is the presence of O in other functional groups (e.g., carbonyl C=O). The C/H ratio is considered as an indicator of aromaticity. Higher values show a lower aromaticity and higher content of single bonds between C atoms.

**Figure 1:**
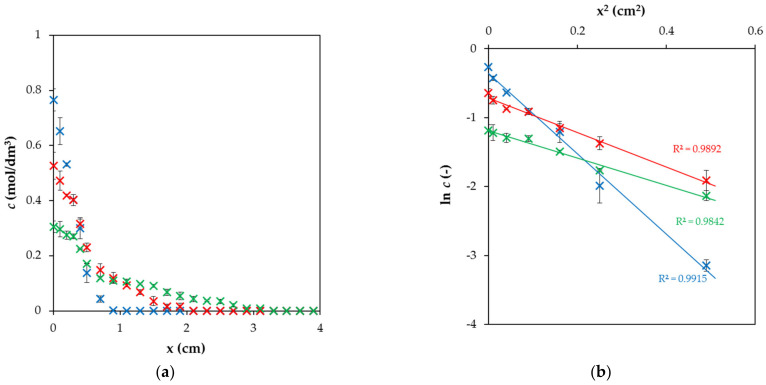
The example of experimental data: (**a**) Concentration profiles in agarose hydrogel enriched by ESHA in different times from the beginning of diffusion (6 h—blue, 24 h—red, 72 h—green); (**b**) The same experimental data linearized according to Equation (2).

In this work, the effect of lignitic humic acids and standard humic acids (purchased from IHSS) on the diffusion of a small amount of Cu(II) ions in agarose hydrogel was studied. Copper(II) is a traditional model metal ion used for the reactivity mapping of humic substances [1,2,39,40,42]. In contrast to previous works [37,38], the method of instantaneous planar source [39,40,41,42,43,44] was used. In [37], diffusion through the layer of hydrogel was studied in the stationary state. The experimental arrangement of donor and acceptor compartments spaced by the hydrogel layer was used. Metal ions passed through the layer and the concentration of metal ions in both compartment was monitored. In [38], hydrogels were saturated by metal ions before the experiment. Humic acids were in equilibrium with copper(II) and the release of metal ions from hydrogels were studied. The difference between previous works [37,38] and the approach applied in this study is that only a small, predefined amount of diffusing particles (metal ions) is applied on the surface of hydrogel. The particles then diffuse into hydrogel and their surface concentration decreases (because the source is exhausted soon). At equilibrium, the homogeneous distribution of metal ions in hydrogel is achieved. It means that the concentration of metal ions is then constant and independent to the distance from interface. The concentration profile in equilibrium is thus the line parallel with the x-axis. In this work, a much lower concentration of metal ions were present in the studied hydrogels and the dependence of diffusivity on the content of diffusing particles in hydrogel was explored. The mathematical model applied on the data is based on the presumption that the diffusion is not closed to equilibrium, and that the diffusion trajectories are shorter that the hydrogel dimension. Simultaneously, the amount of metal ions applied on the interface is known and no metal ions are present in hydrogel at the onset of the experiment. The final solution of second Fick’s second law is [43,44]:

(1) $$c=\frac{n}{S\sqrt{\pi D_{\mathrm{eff}}t}}exp\left(-\frac{x^{2}}{4D_{\mathrm{eff}}t}\right)$$

and after logarithmation

(2) $$\ln c=\ln\frac{n}{S\sqrt{\pi D_{\mathrm{eff}}t}}-\frac{x^{2}}{4D_{\mathrm{eff}}t}$$

where *n* stands for the total mass of diffusing compound applied in the form of a narrow pulse and *S* is the cross–section area available for the transport of the compound. The effective diffusion coefficient *D*_eff_ can then be determined from the slope of the linear regression of $\ln c=f\left(x^{2}\right)$ [43,44]. The experimental data obtained for the hydrogel enriched by ESHA and the data linearized according to Equation (2) are shown in Figure 1. It can be seen that the concentration of Cu(II) ions at interface decreased and they permeated in longer distance into hydrogel with continuing diffusion as expected. Additionally, the experimental data distribution is in a good agreement with Equation (2), based on the value of the coefficients of determination.

The diffusion coefficient *D* for Cu(II) ions in water is tabulated [46] and their value is equal to 1.43 × 10^−9^ m^2^·s^−1^. If metal ions diffuse in hydrogel, their motion is influences by its pore structure, and the diffusion coefficient in hydrogel *D*_g_ is lower than that in water (*D*). If the hydrogel contains a reactive component (humic acids in our case), a portion of the metal ions can be immobilized and an apparent equilibrium constant between immobilized and free movable metal ions (*K* = *c*_im_/*c*_free_) can be included into the effective diffusion coefficient *D*_eff_.

(3) $$D_{\mathrm{eff}}=D\frac{\phi }{\tau \left(K+1\right)}=D\frac{\mu }{K+1}=\frac{D_{g}}{K+1}$$

where the parameter *φ* is the ratio of the effective diffusive cross section, which is available for transport of Cu(II) ions, to the bulk cross section. The available cross section is smaller than in case of a homogenous material because the diffusion takes place only through the fluid-filled pores and voids of humic hydrogels. Because the pores are not straight, the diffusion takes place more effectively over a longer distance than it would in a homogenous material. The tortuosity *τ* is a value characterizing the longer distance traversed in the pores. The parameter *μ* (=*φ*/*τ*) represents the influences of the structure of humic hydrogel and its local geometry in the diffusion [37,38,39,40]. The structural parameter *μ* thus can be calculated as the ratio between the diffusion coefficient of Cu(II) ions in nonreactive hydrogel (agarose hydrogel without humic acids) *D*_g_ and the diffusion coefficient *D* of Cu(II) ions in water *D.* The apparent equilibrium constant *K* can be expressed on the basis of Equation (4) as

(4) $$K=\frac{D_{g}}{D_{\mathrm{eff}}}-1$$

The value of *D*_g_ for pure agarose hydrogel was found to be 3.71 × 10^−10^ m^2^·s^−1^. The values of the effective diffusion coefficient *D*_eff_ as well as those of apparent equilibrium constant *K* are listed in Table 2. The contents of free and immobilized Cu(II) ions were calculated on the basis of the values of apparent equilibrium constant *K*, which is the ratio between immobilized and free Cu(II) ions. We know the total amount of Cu(II) ions in hydrogel (see section Materials and methods) is equal to the sum of these two forms of metal ions. Alongside, we know the ratio of these two forms and the combination of these two relationships resulted in the contents of free movable and immobilized Cu(II) ions in hydrogels.

The values of diffusion coefficients can be compared with results published by other authors. Scally et al. [47] determined Diffusion coefficients of metals and metal complexes in hydrogels based on polyacrylamide the commonly used technique of diffusive gradients in thin films (DGT). Their values for Cu(II) ions ranged between 6.05 and 6.51 × 10^−10^ m^2^·s^−1^ and decreased strongly if metal ions were complexed with humic and fulvic acids. A similar decrease in diffusion rate caused by complexation of metal ions with humic substances and other ligands was observed in [48]. Wang et al. [49] studied diffusion characteristics of agarose hydrogel used in DGT. The diffusion coefficients through the agarose gel (6.59 × 10^−10^ m^2^·s ^−1^ for Cu) were slightly higher than values reported for the agarose cross-linked polyacrylamide. Garmo et al. [50] determined the diffusion coefficients of 55 elements in diffusive agarose polyacrylamide gels of the regular type used in the DGT technique. Their values for copper ranged between 5.5 and 6.6 × 10^−10^ m^2^·s ^−1^. In contrast, the values of diffusion coefficients were lower in magnitude (e.g., 4.75 × 10^−11^ m^2^·s ^−1^ [51] and 2.12–3.12 × 10^−11^ m^2^·s ^−1^ [52]). Our value of *D*_g_ for pure agarose hydrogel is lower in comparison with the diffusion coefficients published in [47,48,49,50]. It is necessary to account for different types of hydrogels and the different methods of the determination of the diffusion coefficient. Results published in [47,48,49,50] were determined using the DGT technique, which is characterized by the diffusion in thin film. The method used in this work belongs to the diffusion in semi-infinite medium, where the diffused particles penetrate into hydrogel and their concentration on the end of hydrogel sample is unchanged during the experiment. The incorporation of humic acids into the hydrogel resulted in the decrease in diffusivity of a magnitude comparable with values published in [51,52].

The above mentioned equations can be used for data processing on condition that the chemical reaction between Cu(II) ions and humic acids is much faster that the diffusion, and the local equilibrium can be assumed to exist between the free and immobilized metal ions. It means that the front of diffusing Cu(II) ions travels through the hydrogel containing non-occupied binding sites. Metal ions thus can interact immediately with humic acids and the quick local equilibrium can be achieved. On the basis of the knowledge of total acidities *β* (see Table 1) and calculated values of apparent equilibrium constant *K*, the content of free movable and immobilized Cu(II) ions in hydrogels can be determined (Table 2).

**Figure 2:**
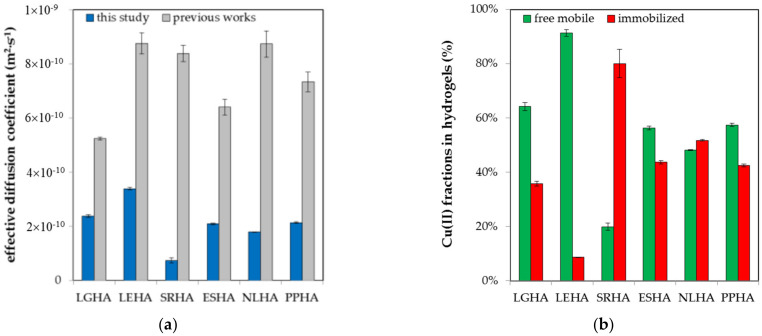
Results of diffusion experiments: (**a**) The comparison of effective diffusion coefficients determined in this study (blue) and previous works (grey); (**b**) The content of free mobile (green) and immobilized (red) fraction of Cu (II) ions in hydrogels enriched by humic acids.

As can be seen, the values of effective diffusion coefficients were found to be of the magnitude ~10^−10^ m^2^·s ^−1^ which corresponds with values published elsewhere [37,38] for metal ions. In comparison with the results obtained for higher amounts of diffusing Cu(II) ions [38], the effective diffusion coefficients determined in this work are lower, being on average ~30% of the previous results with the exception of LGHA (45%) and SRHA (9%) (see Figure 2). These differences between the data reported herein and those previously obtained by different experimental arrangements and mathematical models can be caused by multiple factors. As described above, the structure of humic acids can be characterized by a supramolecular arrangement of relatively small particles [12,16,17,53,54,55] often in co-existence with larger macromolecules [11,16,17,18]. The structure of humic acids is very dynamic and sensitive to the surrounding environment (concentration, pH, ionic strength) [23,24,25,31,41,53]. The incorporation of humic acids into hydrogel can influence its inner structure, including the distribution, size and shape of hydrogel pores [37]. Despite the fact that the content of humic acids is the same for all of the humic samples used and the same as in previous works [34,35,37,38], different humic acids have different structures, different content of binding sites and different molecular arrangements. The supramolecular structure of humic acids is very sensitive to their chemical vicinity, including the presence of reactive metal ions. Metal ions influence not only pH and ionic strength but also can alter the spatial configuration of humic acids. They have more possibilities of interacting with humic acids and coordinating more binding sites into one metal-humic complex. The binding sites can be functional groups belong to one or more humic molecules [23,24].

The most important binding sites in humic molecules are carboxylic and phenolic functional groups [1,2,3,4,5,6,22,32,39,40]. Their different combinations can result in a large number of different coordination (chelation) sites that are able to bind metal ions by attractive interactions of different strengths. The sites can be bifunctional (e.g., salicylic type) or complexes connecting two bifunctional ligands can be formed. A possibility of other types (e.g., three functional groups) can be also taken into consideration. The spatial arrangement of humic acids and the formation of metal-humic complexes are thus strongly affected by the content of metal ions in the hydrogel (or more precisely by the number ratio between metal ions and binding sites in humic acids). It results in different effective diffusion coefficients and (also) apparent equilibrium constants included in the values of *D*_eff_ (see Equation (3)). As mentioned above, the value of *K* is only apparent. This means that the simple equilibrium between free and immobilized ions (Cu (II)_free_ ↔ Cu (II)_im_) does not correspond with the real mechanism of reaction between metal ions and humic acids in hydrogel. One of the differences can be, e.g., the splitting off of a hydrogen ion from acidic functional group during complexation. Therefore, the value of *K* cannot be considered as the true equilibrium constant from a thermodynamic standpoint, but as the ratio between two forms of metal ions in hydrogel (resembling to a partitioning coefficient). The main differences between the results of this study and the values of *D*_eff_ determined by means of other experimental setups and mathematical models are due to the dynamic supramolecular structure of humic acids that are able to react sensitively to changes in their environment and a potential dependence of the diffusivity of metal ions on their concentration. It is also necessary to take into account the influence of metal ions on other conditions of the surrounding environment, such as pH and ionic strength.

By comparing the percentages of free mobile and immobilized fractions of Cu (II) ions in hydrogels, it can be stated that only humic acids isolated from water matrices (SRHA and NLHA) can immobilize more than 50% of metal ions. The immobilized fraction predominates only in the case of SRHA and both fractions are approximately comparable for ESHA, NLHA and PPHA. In contrast, the free mobile fraction predominates in the cases of coal-based humic acids (LGHA and LEHA). With the exception of SRHA, these findings are generally in agreement with the results reported previously [38], where the mobile fraction of Cu (II) ions prevails in all studied hydrogels.

**Figure 3:**
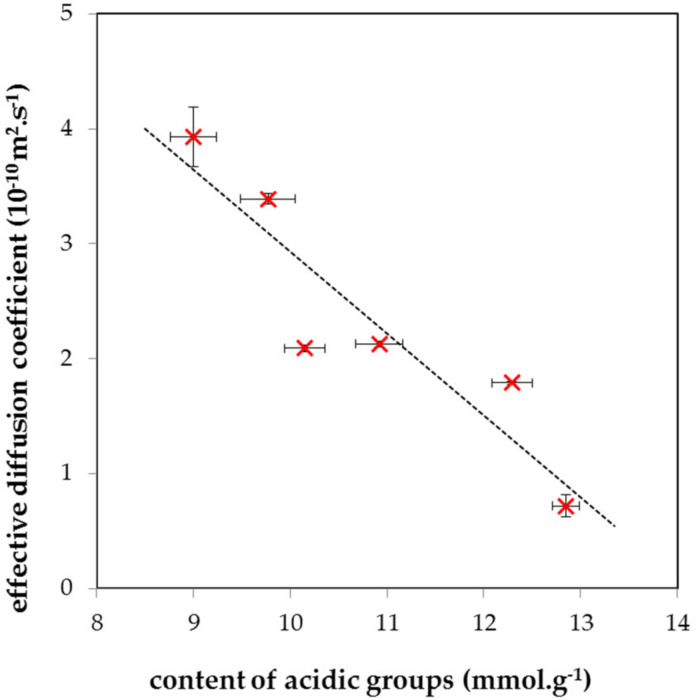
The dependence of effective diffusion coefficient calculated using Equation (2) on the content of acidic groups in humic acids (see β values in Table 1).

Figure 3 shows the dependence of the effective diffusion coefficients on the acidity of the studied humic acids. All hydrogels containing humic acids had lower diffusivity in comparison to pure agarose hydrogel, as expected. The decrease was caused by the high affinity of copper to humic acids and its partial immobilization in hydrogel structure. Previous works showed that the transport of metal ions in humic gels is strongly influenced by the reactivity of humic acids; therefore, changes in the content of acidic functional groups result in changes of diffusivity [39,40,41]. The inverse proportionality between the effective diffusion coefficients and the content of acidic functional groups was observed. It indicates that the increase in the content of acidic functional groups was related to the decrease in the mobility of Cu (II) in hydrogels.

## 3. Materials and Methods

Six different humic acids were used in this work. One sample of humic acids was extracted from lignite mined in the Czech Republic (Mikulčice in South Moravia) and the other samples were purchased from International Humic Substances Society (IHSS, St. Paul, MN, USA) [45].

Lignitic humic acids (in this work designated as LGHA) were isolated following the same procedure used in our previous research [2,22,23,24,25,26] (more details regarding chemical structure and the isolation procedure can be found elsewhere [2,34,35,39,49].

The following humic samples were purchased from IHSS: Nordic Lake Humic Acids 1R105H (NLHA), Elliot Soil Humic Acids 1S102H (ESHA), Suwannee River Humic Acids 2S101H (SRHA), Pahokee Peat Humic Acids 1S103H (PPHA), and Leonardite Humic Acids1S104H (LEHA).

All hydrogels utilized in diffusion experiments were prepared via thermoreversible gelation of aqueous solution of agarose described previously [34]. Agarose hydrogels were gelatinized from the solution of agarose in water or aqueous solutions of humic acids. Dry agarose content in gel was 1 wt.%, dry content of humic acids was 0.01 wt.%. The mixture was slowly heated under continuous stirring at up to 80 °C and maintained at the temperature until the occurrence of the transparent solution. The solution was degassed in ultrasonic bath and slowly poured into the PMMA spectrophotometric cuvette. The cuvette orifice was immediately covered with the pre-heated plate of glass to prevent drying and shrinking of the gel. Gentle cooling of the cuvettes at laboratory temperature led to the gradual gelation of the mixture [34,35,37,38].

A square slice of filtering paper (1 × 1 cm) was sunk into the solution of the 1M CuCl_2_ (1 min) and then added to one top of the cuvette filled with the hydrogel. The amount of Cu (II) ions in the filtering paper was determined as the average of measurements in leachates from ten saturated filtering papers. The amount of Cu (II) ions diffused into hydrogel was calculated as the difference between average Cu (II) content in filtering paper and the residue of Cu (II) ions in the filtering paper after its removing. The cuvette was packed with parafilm and aluminium foil to prevent the hydrogel drying. The durations of the diffusion experiments were 6, 12, 24, 48 and 72 h. In these time intervals, the cuvettes were taken out of the solution and the UV-VIS spectra were recorded considering the distances from the orifice on Varian Cary 50 UV-VIS spectrophotometer (Agilent Technologies, Palo Alto, CA, USA) equipped with the special accessory providing a fine control of the vertical movement of the cuvette in the spectrophotometer. The concentration of Cu (II) ions was determined at different positions in the gels by means of a calibration line. UV-VIS spectra were calibrated for the hydrogels with the known concentration, homogenously distributed in the whole volume of the gel. These hydrogels samples were prepared using exactly the same preparation procedure as for the samples for the diffusion experiments; only the precise amount of the dye was added to the solution before gelatinization. Agarose (AG; routine use class) and CuCl_2_.2H2O (p.a.) were purchased from Sigma-Aldrich (St. Luis, MO, USA). All experiments were performed at laboratory temperature (25 ± 1 °C). Data are presented as average values with standard deviation bars.

## 4. Conclusions

In this work, the influence of humic acids on the transport of Cu (II) ions in agarose hydrogels was studied. It was confirmed that humic acids influenced the diffusivity of metal ions in hydrogels. Diffusion coefficient in pure agarose hydrogel (3.71 × 10^−10^ m^2^·s^−1^) decreased significantly if the hydrogel was enriched by humic acids. The lowest value was obtained for the aquatic SRHA sample, which was also the most active in copper-humic interactions and had the highest content of acidic functional groups. In contrast, a low content of functional groups in LEHA sample resulted in an effective diffusion coefficient comparable with the diffusivity of Cu (II) ions in hydrogel without humic acids. In comparison with some results previously reported, only the small defined impulse of diffusing particles penetrated into hydrogel, which led to lower values of effective diffusion coefficients and a higher or comparable immobilized fraction of cupric ions (excepting LEHA). The diffusion was also influenced by the surrounding environment (pH and ionic strength) as well as the spatial arrangement of humic acids.

## Figures and Tables

**Table 1:** Elemental composition and total acidity (β) of studied humic acids (normalized on dry ash-free samples) [45].

Sample	C (% at.)	H (% at.)	N (% at.)	O (% at.)	C/H	O/C	β (mmol·g^−1^)
LGHA	39.07	38.44	1.08	21.41	1.02	0.55	9.00
NLHA	39.81	35.31	0.74	24.14	1.13	0.61	12.19
ESHA	44.34	33.45	2.71	19.51	1.33	0.44	10.15
SRHA	38.64	37.45	0.74	23.17	1.03	0.60	12.85
PPHA	42.36	34.20	2.38	21.06	1.24	0.50	10.92
LEHA	48.18	33.29	0.80	17.73	1.45	0.37	9.77

**Table 2:** Effective diffusion coefficients of Cu(II) ions in hydrogels enriched by different humic acids (*D*_eff_), apparent equilibrium constant (*K*) and contents of free movable and immobilized Cu(II) ions in hydrogels.

Sample	10^−10^ *D*_eff_ (m^2^·s^−1^)	*K* (-)	Free Cu(II) (μmol·g^−1^)	Immobilized Cu(II) (μmol·g^−1^)
LGHA	2.38	0.56	58.78	32.22
NLHA	1.79	1.08	59.23	63.67
ESHA	2.09	0.78	57.14	44.36
SRHA	0.74	4.02	25.58	102.92
PPHA	2.13	0.66	62.76	46.44
LEHA	3.39	0.10	89.21	8.49

## Data Availability

Experimental data can be provided by author if required.

## Sample Availability

Samples of the compounds are available from the author.
